# Leveraging 16S rRNA data to uncover vaginal microbial signatures in women with cervical cancer

**DOI:** 10.3389/fcimb.2023.1024723

**Published:** 2023-01-19

**Authors:** Ming Wu, Hongfei Yu, Yueqian Gao, Huanrong Li, Chen Wang, Huiyang Li, Xiaotong Ma, Mengting Dong, Bijun Li, Junyi Bai, Yalan Dong, Xiangqin Fan, Jintian Zhang, Ye Yan, Wenhui Qi, Cha Han, Aiping Fan, Fengxia Xue

**Affiliations:** ^1^ Department of Gynecology and Obstetrics, Tianjin Medical University General Hospital, Tianjin, China; ^2^ Tianjin Key Laboratory of Female Reproductive Health and Eugenic, Department of Gynecology and Obstetrics, Tianjin Medical University General Hospital, Tianjin, China

**Keywords:** vaginal microbiota, biomarkers, 16S rRNA, cervical cancer, HPV

## Abstract

Microbiota-relevant signatures have been investigated for human papillomavirus-related cervical cancer (CC), but lack consistency because of study- and methodology-derived heterogeneities. Here, four publicly available 16S rRNA datasets including 171 vaginal samples (51 CC versus 120 healthy controls) were analyzed to characterize reproducible CC-associated microbial signatures. We employed a recently published clustering approach called VAginaL community state typE Nearest CentroId clAssifier to assign the metadata to 13 community state types (CSTs) in our study. Nine subCSTs were identified. A random forest model (RFM) classifier was constructed to identify 33 optimal genus-based and 94 species-based signatures. Confounder analysis revealed confounding effects on both study- and hypervariable region-associated aspects. After adjusting for confounders, multivariate analysis identified 14 significantly changed taxa in CC versus the controls (*P* < 0.05). Furthermore, predicted functional analysis revealed significantly upregulated pathways relevant to the altered vaginal microbiota in CC. Cofactor, carrier, and vitamin biosynthesis were significantly enriched in CC, followed by fatty acid and lipid biosynthesis, and fermentation of short-chain fatty acids. Genus-based contributors to the differential functional abundances were also displayed. Overall, this integrative study identified reproducible and generalizable signatures in CC, suggesting the causal role of specific taxa in CC pathogenesis.

## 1 Introduction

Cervical cancer (CC) remains the fourth most common cancer in women worldwide, with 604,127 new cases in 2020 and more than 341,831 deaths, accounting for nearly 8% of all female cancer-related deaths annually (Sung et al., 2021). This common infection-related neoplasm and its premalignant precursor are caused by high-risk human papillomavirus (HR-HPV) infections. HR-HPV persistence is critical for precancerous lesions and cancers.

Microbiota-relevant biomarkers have been identified and characterized in human diseases related to the gut, liver, brain, and lungs. Epidemiological studies have associated the overrepresentation of vaginal non-*Lactobacillus*-dominant microorganisms with HPV, cervical lesions, and CC (Mitra et al., 2015; Brusselaers et al., 2019; Norenhag et al., 2020; Kovachev, 2020), indicating that such species may be less protective and non-optimal in these conditions. However, little is known about the global changes in the structure of communities colonizing the vagina in CC. Several research groups that utilize sequence-based techniques have found associations between CC and vaginal organisms (Laniewski et al., 2018; Cheng et al., 2020; Xie et al., 2020; Kang et al., 2021). Apart from bacterial vaginosis-related microorganisms, other members such as *Streptococcus*, *Staphylococcus*, *Clostridium*, and *Corynebacterium* may exert key tumor-promoting effects in carcinogenesis (Łaniewski et al., 2020; Manzanares-Leal et al., 2022). Consistent with this view, *Staphylococcus*, *Bacteroides*, and *Clostridium* have been identified as signatures in CC (Fan et al., 2021). The mechanistic underpinnings of these epidemiological relationships, particularly the role of specific members or dysbiosis in driving tumorigenesis, remain to be elucidated. A previous study indicated that the *L. iners* metabolite lactate can activate Wnt signaling through the lactate-Gpr81 complex, which increases the level of core fucosylation in epidermal cells and inhibits the proliferation and migration of CC cells (Fan et al., 2021). However, studies on different cohorts may generate population-specific results. Further, these studies show substantial variations in signatures, largely because of various biological effects influencing the taxonomic profiles of vaginal microbial configuration and inconsistent processing of sequencing data.

Despite the advances in bioinformatics approaches related to microbiota and disease, microbiota research continues to be hindered by methodological challenges. Meta-analysis provides a subset of tools that are powerful and compressive in reducing the effect of methodological and biological confounders resulting in reproducible and rigorous yields across numerous studies (Wirbel et al., 2019). However, informative descriptions of reliable bacterial profiles and CC signatures in meta-analyses with large sample sizes remain lacking. Furthermore, the vaginal microbiota is highly dynamic and prone to physiological, host genetic, and environmental influences (France et al., 2022); therefore, heterogeneities between different studies are inevitable. In a seminal work, France et al. established a VAginaL community state typE Nearest CentroId clAssifier (VALENCIA) tool based on the nearest centroid classification algorithm and leveraged it for assigning vaginal microbiota to community state types (CSTs) (France et al., 2020), which were previously introduced by Ravel et al. (2011) in 2011. VALENCIA harbors a broad application to the vaginal microbiome, with no specificity to disturbed cancer-related communities. This enables cross-study comparisons and facilitates negation of the limitations from previous studies (sample numbers, geographic location, or age), resulting in a global characterization of CC-related vaginal microbiomes from multiple datasets. Overall, combining different datasets to investigate the vaginal microbial patterns correlated to CC based on the VALENCIA method, can help determine the relevant mechanisms of bacterially driven tumorigenesis.

In this study, we present a combined analysis of four studies, including 16S rRNA amplicon sequencing data from 51 CC cases and 120 healthy controls, to investigate the generalizable CC-associated microbial signatures, thus contributing to the overall understanding and interpretation of the vaginal microbiota as a regulator of CC pathogenesis.

## 2 Methods

### 2.1 Data acquisition and preprocessing

We collected vaginal microbiota datasets containing 16S rRNA gene sequencing reads from women with CC and from healthy controls using published literature and the National Center for Biotechnology Information (NCBI) database. The inclusion criteria for the study, were as follows: (1) studies containing publicly available 16S rRNA gene sequences utilizing 454 or Illumina sequencing platforms up to March 31, 2022; (2) studies using vaginal swabs as sample source; (3) women with CC according to the results of HPV testing and histology of cervical biopsy, compared to healthy controls (HPV-negative and cytology-negative women) or HPV-negative women. Considering the anatomical differences and substantial site-associated discrepancies in the microbiome (Kim et al., 2009; Zhang et al., 2021), we restricted the sample type to the vagina. Four cohorts with accessible raw data from vaginal samples were selected for this study (PRJNA687644, SRP122481, PRJNA448161, and PRJNA518153).

Sequence read archive (SRA) files of all samples were downloaded using prefetch software. The 16S raw data were preprocessed using quantitative insights into microbial ecology 2 (QIIME2) (Bolyen et al., 2019). To eliminate sequencing errors, the Divisive Amplicon Denoising Algorithm 2 (DADA2) tool was used to denoise sequencing reads and yield amplicon sequence variants (ASVs) (Wang et al., 2007). Next, the ASVs were aligned and classified *via* the SLIVA 132 database (Quast et al., 2013) for each dataset according to the primers used and the length of the reads; we then applied it to classify the taxonomy for the ASVs in the dataset. Singleton, chloroplast, and mitochondrial ASVs were filtered (Wang et al., 2020). Phylogenetic investigation of communities by reconstruction of unobserved states (PICRUSt2) analysis was conducted using ASVs as the input for each sample (Douglas et al., 2020).

### 2.2 VALENCIA clustering analysis

We employed a novel clustering approach based on the nearest centroid classification algorithm, termed as VALENCIA, for the reproducible and rigorous classification of all samples. Assignments at the species level were based on 13 previously described sub-CSTs. CST I (*L. crispatus* dominated) and III (*L. iners* dominated) are divided into two sub-CSTs, denoted as CST-A and CST-B, respectively, which are more common than CST II (*L. gasseri* dominated) and CST V (*L. jensenii* dominated). The former represents focal species with high relative abundance whereas the latter represents those with lower abundance (France et al., 2020). CST II, including non-optimal microorganisms, consists of CST IV-A, IV-B, and IV-C. Among these, CST IV-A has high relative abundance of *Candidatus Lachnocurva vaginae* (formerly known as BVAB1) and moderate relative abundance of *G. vaginalis*, whereas IV-B shows a contrasting trend. CST IV-C is divided into five sub-CSTs as follows: CST IV-C0—an even community with a moderate amount of *Prevotella*, CST IV-C1—*Streptococcus* dominated, CST IV-C2—*Enterococcus* dominated, CST IV-C3—*Bifidobacterium* dominated, and CST IV-C4—*Staphylococcus* dominated (France et al., 2020). The reference centroids are available at github.com/ravel-lab/VALENCIA. We assigned discordant samples in four different datasets to the CSTs using VALENCIA. We then conducted study- and sequencing target-level comparisons.

### 2.3 Random forest model

To assess the predictive performance of genus-based CC-related signatures, a random forest classifier was trained on 80% of the data and tested on the remaining 20% of our data using the random forest package in R (Breiman, 2001; Breiman, 2002). In order to evaluate the performance of the predictive model and get more precise curves, we used a 10-fold cross-validation within the training set. The cross-validational error curves (average of 10 test sets each) from five trials of the 10-fold cross-validation were averaged. Variable importance by mean decrease in accuracy was calculated for the random forest models using the full set of features. The number of variables was 33 at the genus level and 94 at the species level at the lowest cross-validational error. Thus, the predictive model was constructed using the 33 or 94 most important variables, which were further applied for receiver operating characteristic curve (ROC) analysis.The performance of the models was measured as area under curve (AUC) when applied to the test set, and the confidence intervals for ROC curves were calculated using the pROC R package (Robin et al., 2011).

### 2.4 Multivariate analysis

First, we conducted a cofounder analysis at the genus levels using non-metric multidimensional scaling (NMDS) and permutational multivariate analysis of variance (PERMANOVA). We performed an analysis of the similarity of communities according to the variables relevant to the disease, study and hypervariable regions, and visualize this *via* NMDS in vegan package (Dixon, 2003). The reference are available at https://rdrr.io/cran/vegan/man/metaMDS.html. We measured community differences using Bray-Curtis distance.

Next, we conducted a multivariate meta-analysis to consistently detect differentially changed taxa in CC *vs*. those in the controls across datasets, using the MetaDE software in R (Wang et al., 2012). The statistics (fold-change and FDR *P*-value) for each altered taxon were obtained. The three selected datasets had the same sequencing targets, further alleviating the confounding factors. Additional conservative and biologically concordant findings were obtained using the combined effect size (ES) method for meta-analysis.

Further, we employed PICRUSt2 for the differentially functional profiling of vaginal microbiota from the MetaCyc database (Caspi et al., 2016). Then, based on the predicted functional pathways, we assessed the contribution of the top 30 genera to the distinguished pathways using z-score normalization. The contribution was defined as the proportion of the functional abundance of each genus to the total functional abundance of the top 30 genera in each differential pathway.

### 2.5 Code availability

The codes and scripts are available at https://github.com/yuhongfeilll/Leveraging-16S-rRNA-data-to-uncover-vaginal-microbial-signatures-in-women-with-cervical-cancer.

## 3 Results

### 3.1 Taxonomic composition and VALENCIA clustering

In total, of 51 CC and 120 healthy control samples were acquired from the four 16S rRNA datasets. The demographic and clinical features of these studies are shown in **Table 1**. All samples were sequenced at a suitable depth for further analysis. The count of sequencing reads in samples ranged from 72 to 150,051, with an average of 23,285.

**Table 1:** Characteristics of the 4 datasets included in this study.

Cohorts	Groups	Age (average ± SD)	BMI (average ± SD)	Race	HPV status^a^	Clinical stage	Sequencing platform	Sequencing target	Accession
China-SH (Chen et al., 2020)	Cancer: 9 Control: 68	56.11 ± 9.02 43.00 ± 8.69	23.99 ± 0.68 22.94 ± 2.74	Asian: 77	HPV16/18+: 4 HPV others+: 1 LR-HPV: 4	NA	Illumina MiSeq	V3 -V4	SRP122481
China-GX (Jiang et al., 2021)	Cancer: 20 Control: 6	≤ 54: 10^a^ >54: 10^a^	NA	Asian: 26	HPV16/18+: 15 HPV52/58/59+: 3 HPV-: 2	I B2: 1 II A2: 3 II B: 8 III B: 4 IV A: 3 V B: 1	Illumina HiSeq	V3 -V4	PRJNA687644
USA1 (Tsementzi et al., 2021)	Cancer: 12 Control: 30	NA	NA	white: 23 African-american: 14 Asia: 5	NA	NA	Illumina MiSeq	V3 -V4	PRJNA448161
USA2 (Ilhan et al., 2019)	Cancer: 10 Control: 18	38.90 ± 9.1 40.4 ± 7.0	27.1 ± 7.0 31.4 ± 11.5	Hispanic: 9 non-Hispanic: 19	NA	NA	NA	V4	PRJNA518153

^a^cervical cancer, NA, not available.

**Figure 1:**
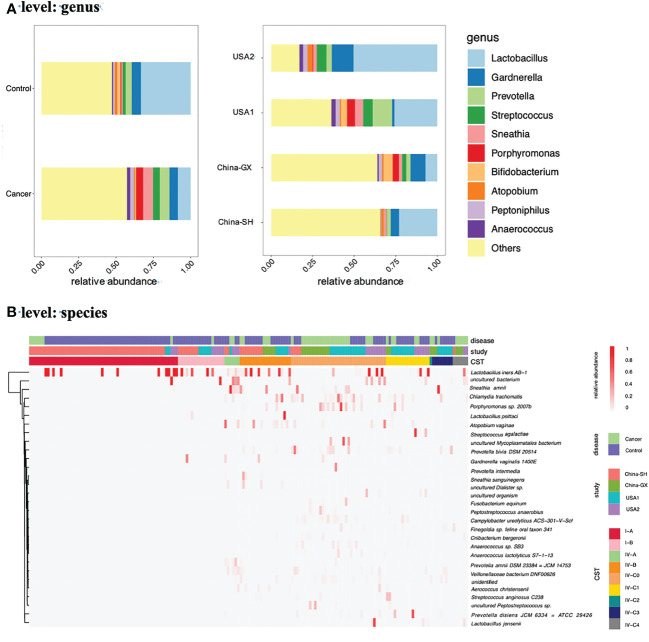
The relative abundance of the top 10 genera and 30 species across all samples. **(A)** Bar chart showed the relative abundances of the top 10 genera according to the disease and study. **(B)** Heatmap showed the relative abundances of the top 30 species across 4 studies. The proportions of samples assigned to each CST across all samples were also presented **(B)**.

At the genus level, 242 taxa were identified in total; among these, the top 10 taxa are displayed in **Figure 1A**. They were as follows: *Lactobacillus*, *Gardnerella*, *Prevotella*, *Streptococcus*, *Sneathia*, *Porphyromonas*, *Bifidobacterium*, *Atopobium*, *Peptoniphilus* and *Anaerococcus*. At the species level, 183 taxa were identified in total; among these, the top 30 taxa are displayed in **Figure 1B**. The ten dominant taxa were as follows: *Lactobacillus iners AB−1*, uncultured bacterium, *Sneathia amnii*, *Chlamydia trachomatis*, and *Porphyromonas* sp. *2007b*, *Atopobium vaginae*, *Prevotella bivia DSM 20514*, uncultured Mycoplasmatales bacterium, *Prevotella disiens JCM 6334 = ATCC 29426* and *Lactobacillus psittaci.*

Next, we used VALENCIA-based clustering to assign the metadata to the 13 sub-CSTs. In total, nine sub-CSTs were defined. Across all samples, the most common community was CST I-A (58, 33.92%), followed by CST IV-C0 (37, 21.64%), CST IV-B (20, 11.70%), CST I-B (18, 10.53%), CST IV-C1 (17, 9.94%), CST IV-C3 (8, 4.68%), CST IV-A (6, 3.51%), CST IV-C4 (6, 3.51%), CST IV-C2 (1, 0.58%) (**Figure 1B**).

**Figure 2:**
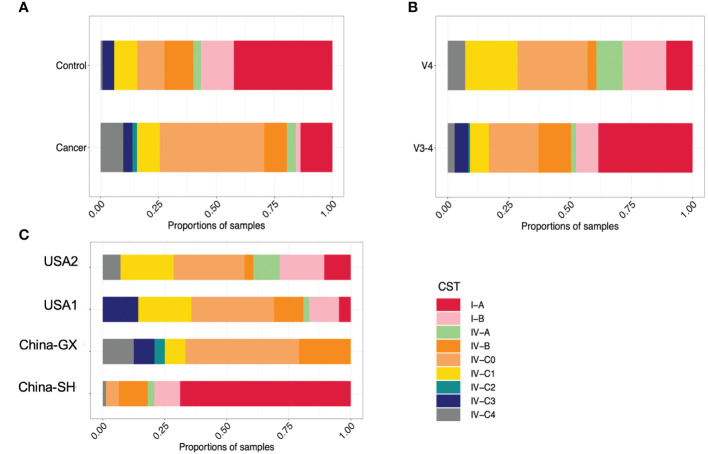
The proportions of samples assigned to each CST in samples grouped by disease **(A)**, study **(B)** and hypervariable regions **(C)**.

Furthermore, in CC, the most common community was CST IV-C0 (23, 45.10%), followed by CST I-A (7, 13.73%), CST IV-B (5, 9.80%), CST IV-C1 (5, 9.80%), CST IV-C4 (5, 9.80%), CST IV-A (2, 3.92%), CST IV-C3 (2, 3.92%) CST I-B (1, 1.96%), CST IV-C2 (1, 1.96%) (**Figure 2A**).

Given the variances in both study- and hypervariable region-related aspects, we also examined the relationships between VALENCIA-defined CSTs and confounders. Examining the distribution of each sub-CST among cohorts and hypervariable regions indicated clear clustering by study and hypervariable region (**Figures 2B, C**). The proportion of sub-CSTs differed among studies and between hypervariable regions (**Supplementary Table 1**).

### 3.2 Identification of vaginal microbial signatures for CC

**Figure 3:**
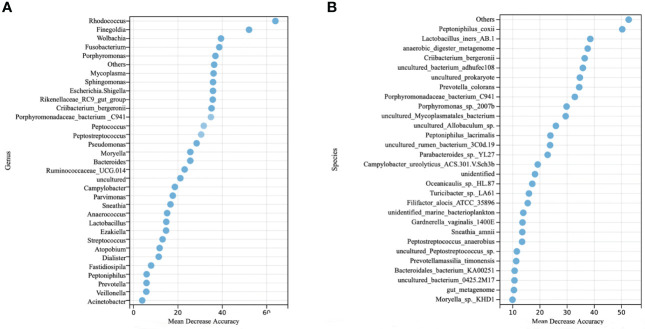
Identification of microbial genus- and species- based signatures of cervical cancer. **(A)** Thirty-three genera were selected as the optimal signatures panel by random forest models between 51 cervical cancer and 120 controls *via* a ten-fold cross-validation. **(B)** The top 30 optimal species signatures panel were presented.

To examine the predictive value of classification based on genus- and species- levels CC-related microbial signatures, a RFM was employed to discriminate the CC from controls. At the genus level, our results showed that the top 33 taxa were selected as the optimal signature sets between the 51 CC cases and 120 controls (**Figure 3A**). The top 33 taxa with mean decreased accuracy of the genera are displayed in **Figure 3A**. Among these, *Rhodococcus* was the highest-ranking signature in this model. Additional signatures included *Finegoldia*, *Wolbachia*, *Fusobacterium*, *Porphyromonas* and so on. High accuracy was achieved based on these 33 signatures, as indicated by an AUC of up to 82.06% (95% CI, 73.72%−91.48%) on the training set when differentiating between the CC and controls with high sensitivity and specificity (**Supplementary Figure 1A**). Interestingly, a good performance was also observed on the test set with an AUC value of 80.73% (95% CI, 64.91%− 96.55%) (**Supplementary Figure 1B**).

At the species level, the top 94 taxa were selected as the optimal signature sets. The top 30 taxa with mean decreased accuracy of the genera are displayed in **Figure 3B**. High accuracy was achieved based on these 94 signatures, as indicated by an AUC of 81.25% (95% CI, 72.35%−90.15%) on the training set (**Supplementary Figure 1D**), and 78.82% (95% CI, 62.17%−95.47%) on the test set (**Supplementary Figure 1E**).

### 3.3 Meta-analysis of the vaginal microbiota in CC versus controls

**Figure 4:**
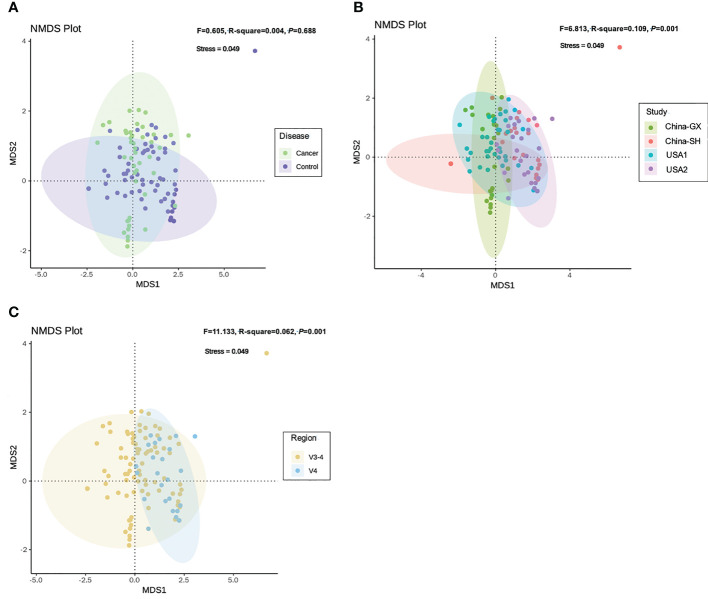
Non-metric multidimensional scaling analysis for all 16S rRNA samples based on genus-level according to the variables relevant to the disease **(A)**, study **(B)** and hypervariable region **(C)**. Adonis test indicated the explained variances with respect to cervical cancer and other variables.

As for the samples between diseases and controls, NMDS revealed larger variance at the genus level (Adonis *F* = 0.605, DF = 1, R^2^ = 0.004, *P* = 0.688; **Figure 4A**; **Supplementary Table 2**). The confounders related to both the study and sequencing target also presented a close relation with the alteration of community dissimilarity. Community dissimilarity based on NMDS showed that the samples were significantly separated by study (*F* = 6.813, DF = 3, R^2^ = 0.109, *P* = 0.001; **Figure 4B**,;**Supplementary Table 2**) and hypervariable region (*F* = 11.133, DF = 1, R^2^ = 0.062, *P* = 0.001; **Figure 4C**; **Supplementary Table 2**). Homogeneity of community dispersions test indicated that the variances between different samples from CC were higher than the samples from the controls (Betadisper *F* = 1.786, *P* = 0.169; **Supplementary Figure 2A**; **Table 3**). Significant differences in community variability were observed with respect to study (*F* = 6.511, *P* = 0.001; **Supplementary Figure 2B**; **Table 3**) and hypervariable region (*F* = 8.219, *P* = 0.006; **Supplementary Figure 2C**; **Table 3**).

**Figure 5:**
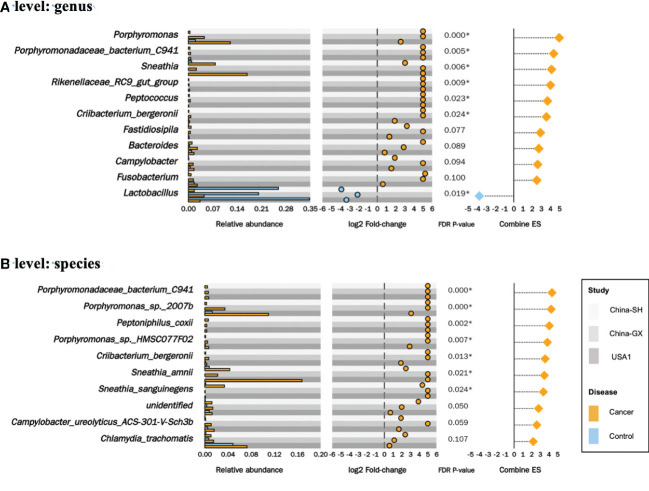
Significantly changed taxa in cervical cancer. The relative abundances in cervical cancer versus controls for the 6 genera **(A)** and 7 species **(B)** significantly enriched and 1 genus decreased **(A)** in the meta-analysis, along with their log2 fold-changes in the 3 datasets, combined effect sizes.

Regarding the impact of the study and sequencing target, we conducted a multivariate analysis to adjust the study- and sequencing target-related factors and identify distinguished taxa at the genus and species levels. In total, 14 distinguishable changed taxa were identified in the meta-analysis of the CC versus controls, including 13 enriched taxa and 1 decreased taxon. At the genus level, six increased taxa (genera *Porphyromonas*, *Porphyromonadaceae_bacterium_C941*, *Sneathia*, *Rikenellaceae_RC9_gut_group*, *Peptococcus* and *Criibacterium_bergeronii*) and one decreased taxon (the genus *Lactobacillus*) were observed (*P* < 0.05, **Figure 5A**). At the species level, seven increase taxa (species *Porphyromonadaceae_bacterium_C941*, *Porphyromonas_sp._2007b*, *Peptoniphilus_coxii*, *Porphyromonas_sp._HMSC077F02*, *Criibacterium_bergeronii*, *Sneathia_amnii* and *Sneathia_sanguinegens*) were observed. (*P* < 0.05, **Figure 5B**).

### 3.4 Predicted functional profiles in CC

**Figure 6:**
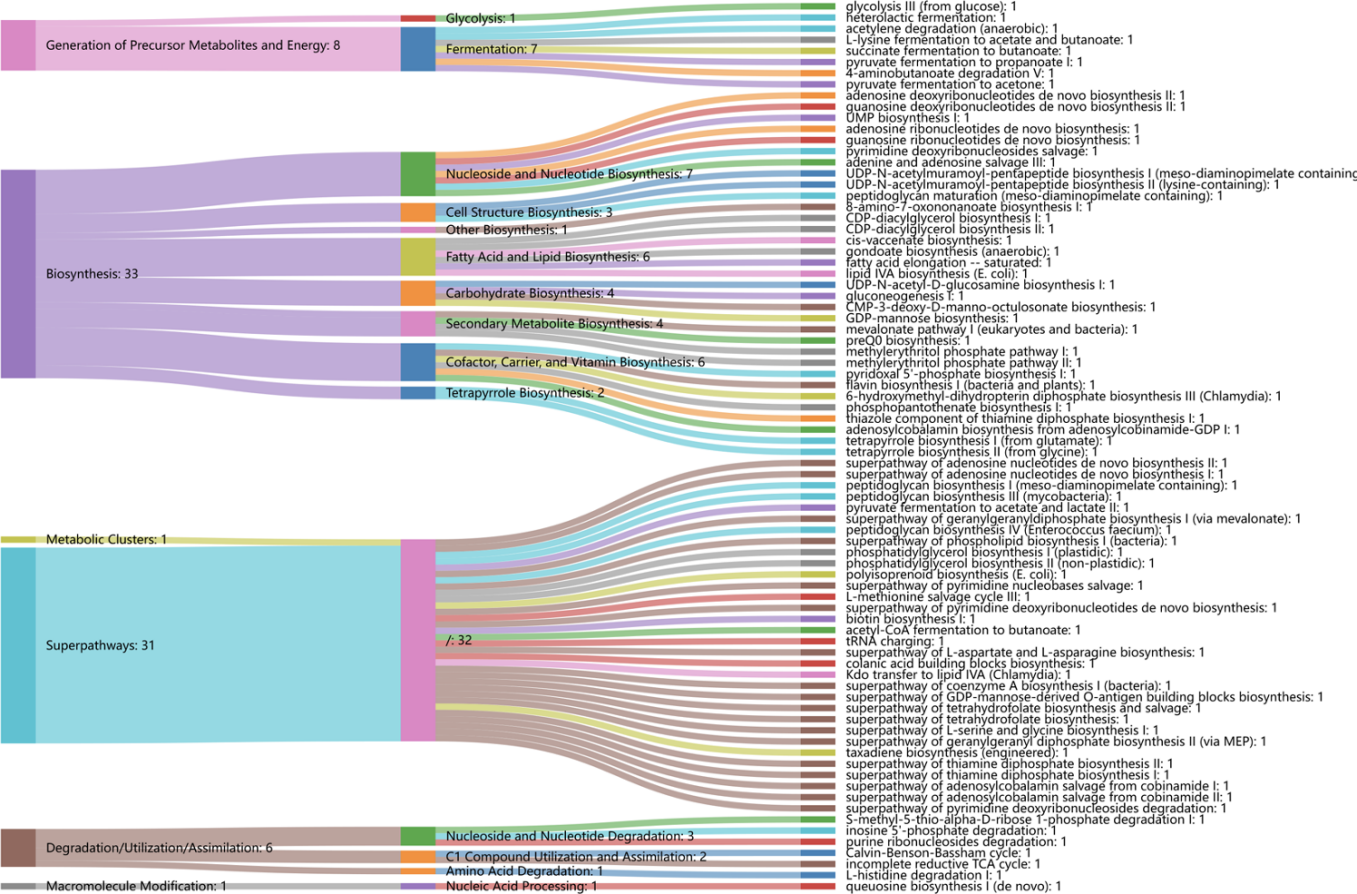
Differentially functional alternations in cervical cancer and controls. Sankey diagram displayed the associations between the differential functional pathways and metabolic reactions. Metabolic reactions were grouped by the MetaCyc pathway categories.

**Figure 7:**
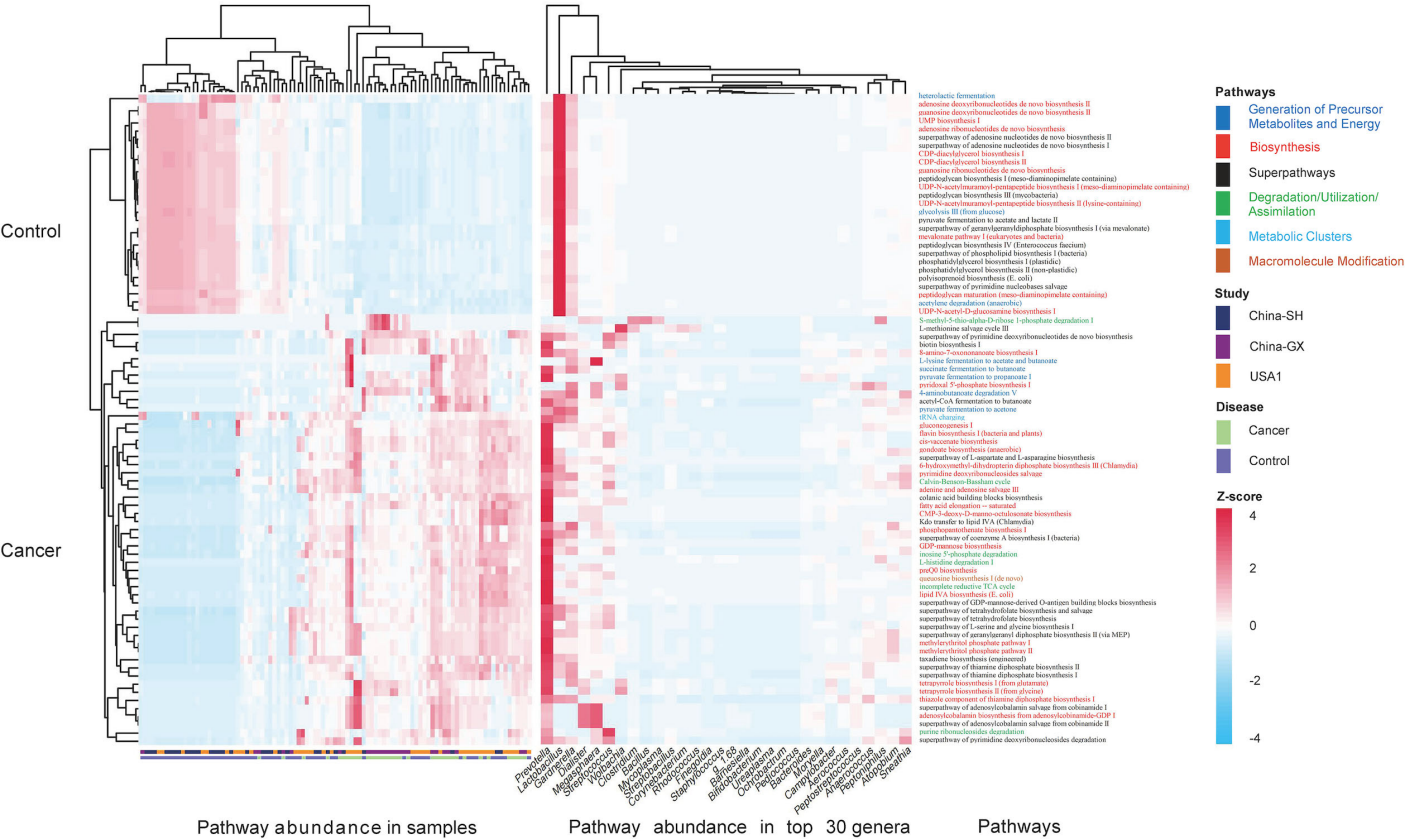
The contributions of the top 30 genera to the differential functional pathways. Heatmap showed the differential functional abundances in cervical cancer and controls (*P*<0.05), and the contributions of the top 30 genera to the pathway abundances. The z-score of each genus to each pathway was indicated in the heatmap. A positive z-score means that the pathway by the genus contributes to its relative increase in cervical cancer, whereas a negative z-score means the pathway by the genus contributes to its decrease in cervical cancer.

Regarding functional profiles, we further assessed the microbiome-related functional profiles and the contribution of genus-based taxa to distinguish functional pathways. In total, we examined 80 differentially enriched pathways between CC and controls, with 53 and 27 differentially enriched pathways in CC and controls, respectively. Specifically, cofactor, carrier, and vitamin biosynthesis were significantly elevated in CC, followed by fatty acid and lipid biosynthesis (i.e., unsaturated fatty acid biosynthesis and lipid IVA biosynthesis), and fermentation (i.e., fermentation to short-chain fatty acids). Carbohydrate biosynthesis (GDP-mannose biosynthesis), nucleoside and nucleotide biosynthesis, and secondary metabolite biosynthesis were also significantly enriched in CC (**Figure 6**). Regarding the contribution of genera to distinguished pathways, *Prevotella* was the only contributor to most functional abundances. Other anaerobes, such as *Gardnerella*, *Dialister*, and *Megasphaera*, were the main contributors to the cofactor, carrier, and vitamin biosynthesis; *Gardnerella*, *Megasphaera*, and *Lactobacillus* were the main contributors to the fermentation of short-chain fatty acids (SCFAs); *Streptococcus* was the main contributor to fatty acid and lipid biosynthesis, along with *Prevotella* (**Figure 7**).

## 4 Discussion

We combined four 16S rRNA datasets to conduct a meta-analysis, including amplicon sequencing data from 51 CC cases and 120 controls. We identified reproducible and generalizable CC-related microbial signatures using a random-forest model. After adjusting for cofounders, multivariable analysis in terms of microbial composition and function revealed significantly altered taxa and pathways. Analysis of the bacterial-based contribution to altered functional abundances elucidated the microbiota-driven regulators in CC pathogenesis.

The RFM has been widely used to identify biomarkers in the field of microbiome research, including colorectal adenoma, CRC, lung cancer, and hypertension (Li et al., 2017; Thomas et al., 2019; Wang et al., 2020; Yang et al., 2021; Liu et al., 2021; Wu et al., 2021). A recent 16S rRNA meta-analysis employed RFM to distinguish colorectal adenomas from controls with an average AUC of 0.80 and to distinguish colorectal adenomas from controls with an average AUC of 0.89 (Wu et al., 2021). We constructed a RFM classification capable of discriminating CC from controls with a good performance under AUC values of 82.6% and 80.73% on the training and test datasets at the genus level, respectively. Similarly, at the species level, this classifier achieved AUC values of 81.25% and 78.82%, respectively. Importantly, these impressive performances were independent of both study- and methodology-associated effects. Confounder analysis indicated that study- and hypervariable region-related factors significantly affected microbial configuration. These findings imply the existence of shared CC-associated biomarkers that can be identified using the RFM classifier, despite the heterogeneities. This further demonstrated that the microbial signatures identified using the RFM were explained by disease status rather than confounder-related aspects.

The vaginal microbial configuration varied between the CC and control groups. The reported altered taxa in CC include BV-related anaerobes and aerobic bacteria (Laniewski et al., 2018; Fan et al., 2021; Wang et al., 2021; Manzanares-Leal et al., 2022). Moreover, the vaginal microbiota in CC were found to present larger amounts of gut bacteria, such as *Streptococcus*, *Peptostreptococcus*, *Enterococcus*, *Escherichia*-*Shigella*, *Staphylococcus*, and *Klebsiella* (Zheng et al., 2019; Wang et al., 2021), which are rare in the vagina under normal conditions, but are common in the gut (Zheng et al., 2019). We observed that both differential taxa and signatures for differentiating CC from controls varied greatly. Some of the differentially elevated bacteria were biomarkers as well at both genus and species levels, such as the genera *Sneathia*, *Peptococcus*, and *Porphyromonas* and the species *Sneathia_amnii* and *Porphyromonas_sp._2007b*. It has been demonstrated that *Sneathia* is the only bacterium overrepresented in the initiation and progression of cervical carcinogenesis and arises as a consequence of the disease (Laniewski et al., 2018). This genus has also been reported as a biomarker in previous observations on cervical lesion and CC (Laniewski et al., 2018; Chen et al., 2020; Mitra et al., 2021). Notably, in our differential abundance analyses, *Sneathia* and *S. amnii* showed significantly increased abundances in CC. This phenomenon may be explained by the potential release of toxic products by adherent *Sneathia* to alter the characteristics of host tissue and directly mediate the effects on the cervical microenvironment (Gentile et al., 2020; Theis et al., 2021). Łaniewski et al. (Laniewski and Herbst-Kralovetz, 2021) demonstrated that *S. amnii* showed the high proinflammatory potential through induction of cytokines, iNOS, and oxidative stress-associated compounds. It is plausible to postulate that this species may contribute to a milieu that favors cervical carcinogenesis by achieving a proinflammatory environment as a regular. The role of this species in CC pathogenesis thus warrants further investigation.

The role of cancer-related microorganisms has been widely discussed in cancer microbiome research, as they provide differential markers for cancer (i.e., diagnosis, causality, and treatment) (Sholl et al., 2022); for instance, two Korean studies identified specific biomarkers to predict the severity of cervical diseases using a RFM (Lee et al., 2020; Kang et al., 2021). Regarding the causal links between CC and vaginal microbiota, it is plausible to postulate that the vaginal microbiota may act as a cancer regulator during cervical tumorigenesis. Functional analysis can provide new insights into the potential bacteria-driven mechanisms and strengthen the interpretation of vaginal microbiota-based carcinogenesis. In particular, evidence has indicated that fatty acids contribute to gynecological carcinogenesis (Mozihim et al., 2022). Fatty acid and lipid alterations in cervicovaginal lavages and blood samples are reported to be closely related to CC (Ilhan et al., 2019; Nam et al., 2021). Lipids that distinguish CC from healthy controls show a strong positive correlation with genital inflammation (Ilhan et al., 2019). A dysbiotic microbiota dominated by aerobic microbes is a potential contributor to an inflammatory microenvironment (Manzanares-Leal et al., 2022). It can also drive pathology by promoting immune evasion that favors tumor cell survival. The genera *Prevotella* and *Streptococcus* were the main contributors to fatty acid and lipid biosynthesis and were also identified as potential microbial signatures in CC. *In vitro* studies have shown that *Prevotella* induces higher concentrations of cytokines compared with *L. crispatus* (Dabee et al., 2021). *P. bivia* is thought to be the major contributor to lipopolysaccharide (LPS) concentrations in vaginal secretions (Dabee et al., 2021). Consistent with this hypothesis, *Prevotella* was found to contribute the most to the biosynthesis of lipid IVA, a key intermediate in LPS biosynthesis (Kovach et al., 1990). LPS activates nuclear factor-kappa B (NF-κB) signaling by binding to Toll-like receptor (TLR) 4 and CD14 on genital epithelial cells, monocytes, and macrophages (Nasu and Narahara, 2010). *Streptococcus*, another contributor, may exert pro-inflammatory effects by producing specific metabolites. Vaccenate discriminated between CC and control cases (Ilhan et al., 2019). In our study, unsaturated fatty acid and lipid biosynthesis (i.e., oleate/vaccenate and gondoate) were significantly upregulated in CC, which is consistent with previous findings. *In vitro* studies have shown that oleic acid can promote the growth of CC cells by upregulating the Src/ERK pathway (Yang et al., 2018). Species from the genus *Streptococcus* (*Group B Streptococcus*) have been reported to be involved in oleic acid biosynthesis during aerobic growth and contribute to virulence (Yamamoto et al., 2006). This suggests that increased *Streptococcus* may be involved in the disease-promoting effects of oleate/vaccenate, thus enhancing genital inflammation and promoting cervical carcinogenesis.

This study has several limitations. First, we conducted a meta-analysis of 16S rRNA data based on a relatively moderate sample size with no identification of taxonomic and metabolic microbial marker genes, thus necessitating comparisons across studies *via* combined metagenomic analysis. The reasons driving the relatively small sample size are multifactorial. For example, we performed a combined analysis of the vaginal microbiome based on knowledge of anatomic potential and substantial site-linked differences in the microbiome (Kim et al., 2009; Zhang et al., 2021). Further, some authors were reluctant to share raw sequences, and several studies lacked complete raw sequences or metadata. Second, despite examining potential confounders such as study and hypervariable regions, demographic-related confounding effects such as age, HPV genotyping, and clinical state of the cancer, were not included in this study. The intricate relationship between the microbial findings and these clinical features was also not observed due to limited raw data.

In summary, our study aggregated and integrated microbial vaginal samples in a consistent pattern from multiple datasets, identified community structures designated using VALENCIA clustering, and characterized bacterial signatures that were uniformly over-represented in CC versus controls among cohorts. The altered microbial taxa and functions established in this meta-analysis could pave the way for further bacteria-driven causal studies on CC. Considering disease- and individual-correlated confounders using multi-omics approaches is thus of great importance. Definitive causality for the key players of unique taxa in CC pathogenesis will emerge from more molecular-relevant epidemiological relationships on a larger scale, as well as clarification of the mechanisms involved in both clinical studies and experimental animal studies.

## Data availability statement

The datasets presented in this study can be found in online repositories. The names of the repository/repositories and accession number(s) can be found in the article/**Supplementary Material**.

## Author contributions

FX contributed to conception and design of the article. MW conducted literature search and drafted the first manuscript. HY and YG performed the data extraction. HuaL, CW, HuiL, XM, MD, BL, JB, YD, XF, JZ, YY, WQ, CH, AF made substantial contributions to revising the article. All authors contributed to the article and approved the submitted version.
