# Supplementary figures and images for: Leveraging 16S rRNA data to uncover vaginal microbial signatures in women with cervical cancer
Source: Front Cell Infect Microbiol. 2023 Jan 19;13:1024723. doi: 10.3389/fcimb.2023.1024723 (PMC9892946; doi:10.3389/fcimb.2023.1024723)

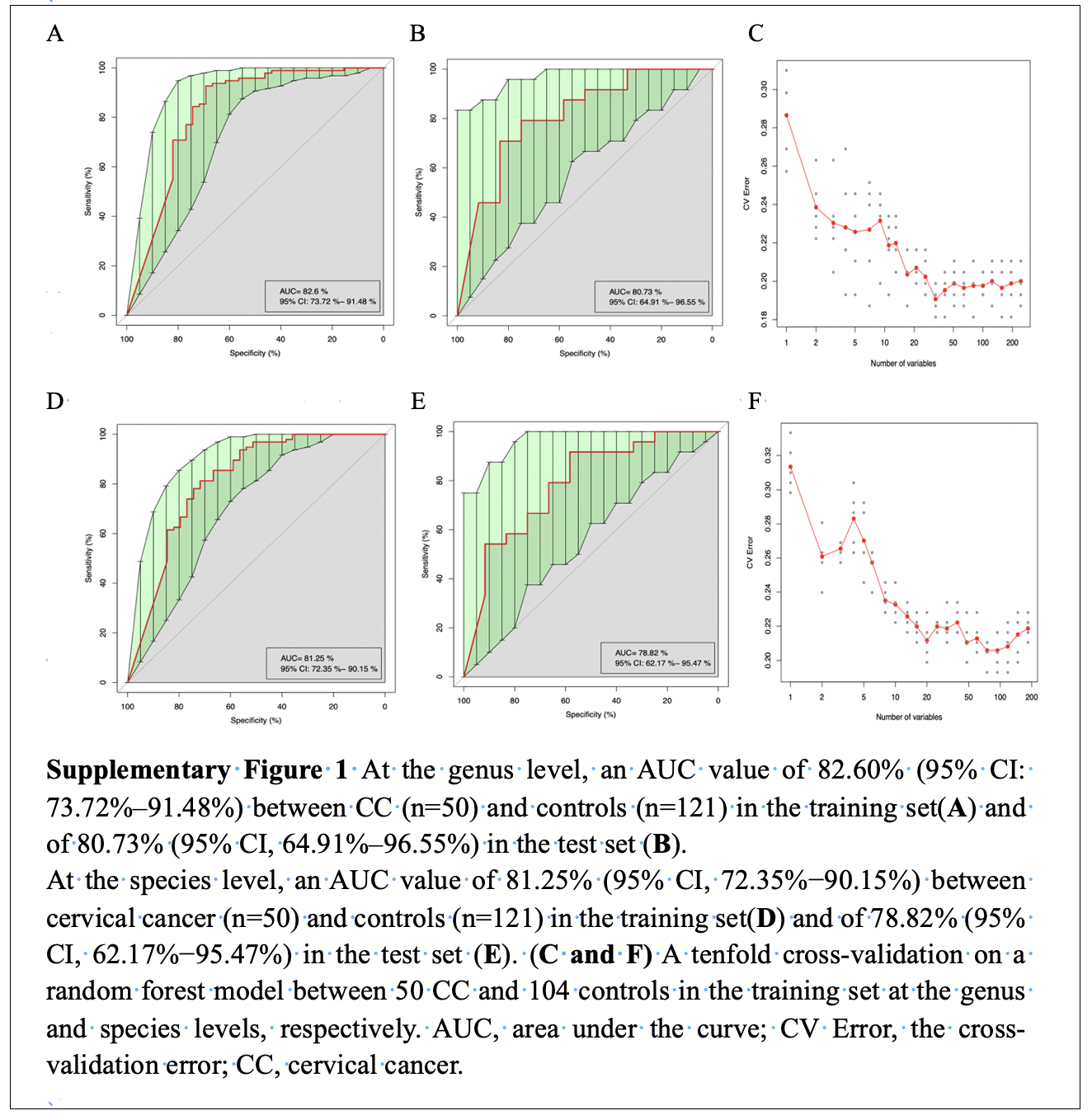

Supplement: Supplementary file 1 [file Image_1.tiff]

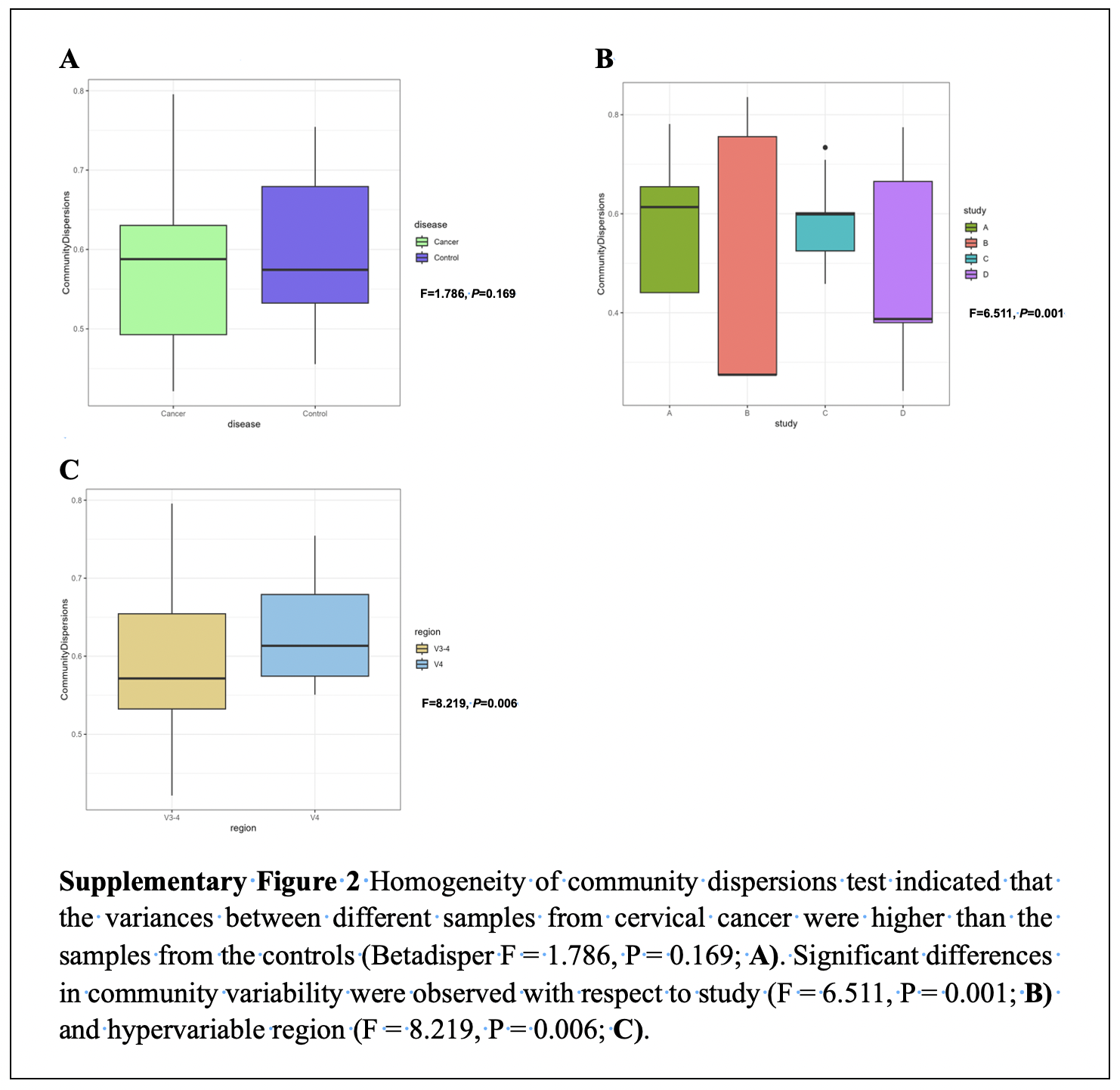

Supplement: Supplementary file 2 [file Image_2.tiff]
